# Enhancing patient satisfaction and reducing nurse workload: the impact of multimedia health education in a prospective single-center randomized controlled trial

**DOI:** 10.3389/fmed.2025.1400061

**Published:** 2025-02-19

**Authors:** Liping Yang, Zilan Qin, Jia Yao, Huan Xu, Jinhui Tian, Yanxian Ren, Haiping Wang, Wenbo Meng

**Affiliations:** ^1^Department of General Surgery, The First Hospital of Lanzhou University, Lanzhou, Gansu, China; ^2^The First Clinical Medical School of Lanzhou University, Lanzhou, Gansu, China; ^3^School of Nursing, Lanzhou University, Lanzhou, Gansu, China; ^4^Gansu Province Key Laboratory of Biological Therapy and Regenerative Medicine Transformation, Lanzhou, Gansu, China; ^5^Evidence-Based Medicine Centre, Lanzhou University, Lanzhou, Gansu, China

**Keywords:** multi-media health education, surgical patients, workload of nurses, patient’s satisfaction, anxiety level

## Abstract

**Introduction:**

Health education is an important part of nursing care. Verbal health education is a common practice in surgical wards, which is time-consuming and laborious. Thus, this study aims to evaluate whether multimedia health education reduces nurses’ workload without compromising patient and family satisfaction in a surgical context.

**Methods:**

We conducted a parallel-group, prospective randomized controlled trial at the Hepatobiliary Surgery Institute of Lanzhou University’s First Hospital between July 2019 and May 2022. Eligible patients (≥18 years) with general surgical conditions and acceptable for surgery were randomly assigned (1:1) to receive a multi-media health education group or a standard health education group. Randomization was performed by an independent statistician using a computer-generated randomization list. The nurses’ workload and satisfaction were the main outcomes; the anxiety level of patients and the variables affecting nurse workload were the secondary outcomes.

**Results:**

A total of 184 eligible participants were randomly assigned to receive multimedia health education and standard health education. The results showed that multimedia health education can shorten the time [15.21 (0.63)vs.16.94 (3.96)] nurses spend on health education during patient admissions, the difference being statistically significant (*p* < 0.001), but it did not lower the satisfaction levels of nurses [73.46 (2.36) vs. 67.16 (5.52)], patients [53.35 (2.09) vs. 47.86 (5.00)], their families [53.35 (2.28) vs. 47.86 (4.53)] and doctors [73.33 (2.40) vs. 68.07 (4.92)] regarding health education (*p* < 0.001); in fact, it increased their satisfaction.

**Conclusion:**

Multi-media health education could reduce nurses’ workload and enhance patient satisfaction but not increase complications.

**Clinical trial registration:**

https://www.clinicaltrials.gov/, identifier [NCT03989401].

## Introduction

Health education has become one of the most important aspects of nursing care. Nurses need to possess a wealth of medical knowledge to effectively execute health education while also fostering patient cooperation for treatment and care. Various studies (1, 2) have shown that surgical procedures often cause severe physical and mental stress to patients. This is the reason why many medical experts and clinical psychologists all over the world are interested in health education for perioperative patients (3). The fear, anxiety, and pain that patients experience during surgery, as well as other complications brought on by surgical trauma, can be significantly reduced by high-quality health education (4, 5).

Additionally, a significant component of nursing work in the medical management of patients is health education (6). Health education methods that address the perioperative needs of patients can not only pique their interest in the subject but also aid them in understanding the disease and receiving training in the healing process. This is crucial for the healing and also to avoid further complications (4, 7–9).

Currently, health education is primarily delivered in the department of surgery through face-to-face verbal guidance and communication, which is time-consuming and labor-intensive. Studies have shown that the increase in nurse workload not only poses a potential threat to nursing quality, but is also closely related to patient mortality, rescue failure rate, and hospital stay (10). Additionally, verbal instruction is influenced by the nurse’s capacity for expression and acknowledgment (3, 6), and because patients vary in terms of age, gender, education level, emotional state, and physical conditions, their responses to the health education are different.

Multimedia health education is a health education model combined with audio-visual stimulation and patients’ participation; it uses the combination of images, words and explanations to make it simpler for patients to understand and also makes health education knowledge more systematic and complete, avoids omissions, addresses patient concerns, and makes health education work procedural, standardized and specific (4, 5, 9, 11, 12). Multimedia health education can expand the scope of information dissemination, making it easier for patients and their families to access more health information. Audio-visual materials are characterized by scientific accuracy, engaging content, timeliness, practicality, and readability, which encourage patients to watch and learn. Moreover, these materials enable patients to relate the information to their own conditions and behaviors, thereby enhancing the effectiveness of health education (4, 5, 8, 9). Some studies (4, 5, 11, 12) have shown that multimedia health education can enhance patients’ and family members’ understanding of perioperative care, promote adequate preoperative preparation, and reduce the incidence of surgical cancelations. Additionally, preoperative multimedia education contributes to positive surgical outcomes, significantly alleviates patient anxiety, improves satisfaction with medical services, and effectively reduces postoperative pain (13–15). Although there are some comparatively effective health education strategies (11–15) that are helpful for the management of perioperative patients, prevention of perioperative complications. Multimedia health education can enhance the effectiveness of verbal health education, making it easier for patients to consolidate, understand, and retain the educational content while reducing the time and frequency required for nurses to deliver education (14–17). However, whether this approach can be both easily and profoundly accepted by patients has become a topic worth exploring in the field of nursing.

In this prospective, single-center, randomized controlled trial, we aim to evaluate whether the application of multimedia health education reduces nurses’ workload without compromising the satisfaction of surgical patients and their families in the surgical context.

## Materials and methods

### Ethical approval

The ethics committee of the First Hospital of Lanzhou University gave its approval to the research protocol (registry number: LDYYLL2019-203). Written informed consent was given by each patient or their legal representative. The trial was registered prior to patient enrollment at clinicaltrials.gov (NCT03989401, Principal investigator: Jia Yao, Date of registration: July 3, 2019).

### Study design and participants

This study, which was carried out at the Hepatobiliary Surgery Institute of Lanzhou University’s First Hospital, was a prospective randomized controlled trial with two arms (intervention group and control group), the intervention group received multimedia health education, while the control group received face-to-face oral health education. The following criteria were required for inclusion: (1) age between 18 and 75 years old; (2) general surgery diseases requiring surgical treatment; (3) education history of at least primary school level; (4) patients with clear awareness, be able to cooperate clinical data collection; (5) adequate communication ability; and (6) informed consent obtained. As exclusion criteria, we determined that patients with visual or hearing impairment, mental illness, dementia or other mental disorders, cardiac, brain or nephropathy complications, those unable to care for themselves, emergency or critically ill patients, and participants of prior studies would be excluded from the study.

### Randomization

Patients were randomly assigned (1:1) to the multimedia health education intervention group or the control group (oral health education). An impartial statistician used a computer generated randomization list to perform the randomization. Office nurses used a series of sequentially numbered, sealed, and opaque envelopes to implement allocation. After the participants had finished the baseline assessment, the envelope was opened in front of them, and the patients were informed of their assignment. Satisfaction was assessed by clinical nurses who know the patient distribution.

### Interventions

#### Multimedia health education

Except for multimedia health education, the participants in the intervention group received the same level of clinical care as those in the control group (standard general surgical nursing care). After the patient has finished the admission process, the clinical responsible nurses have brought patients and/or their family members into the ward, explained how to use the multimedia video player for health education, and turned on the television to play the video on admission. Other wards could not hear the video sound. The following topics are covered in the health education video: (1) hospital and ward environment; (2) primary clinical staff; (3) safety precautions; (4) preoperative planning; (5) visiting, ward round and leave system; (6) regular medical checkups and precautions; (7) dietary advice and precautions; (8) rehabilitation advice and precautions; (9) issues with medication knowledge and patient identification (4, 5, 9, 18–20). The health education video was filmed and produced by our research team in the research department. Nurses and doctors from the research team played the roles of doctors, nurses, and patients in the video, which has a total duration of 14 min. Additionally, the participants and their families should be advised to consult doctors or nurses if they have any doubts.

#### Standard health education

At the time of admission, trained clinical nurses conducted a face-to-face verbal health evaluation with the control group. The verbal health education had the same information as the video did, and the nurses also advised the participants to speak with the doctor or nurse if they had any questions.

Each participant who was placed in wards with only one bed was instructed not to share their admission health education with other patients in other wards in order to minimize the possibility of cross-contamination between the two groups of the trial. In addition, patients and their families can watch the video repeatedly.

### Outcomes

The main outcomes were the workload of nurses on admission (the duration of health education per nurse, times of health education of nurse, inquiry times on health education content of patients and family members on the day of admission) and the satisfaction of patients, families, doctors and nurses with health education. Patients, family members, doctors and nurses were required to fill in the satisfaction questionnaires within 24 h after admission.

The level of patient anxiety before and after receiving health education was a secondary outcome. Additional monitored parameters include the incidence of catheter slippage and pressure ulcers.

### Statistical analysis

Based on the results of the prior studies (19, 20), the satisfaction of the intervention group is 98.22 ± 1.15, while the satisfaction of the control group is 97.03 ± 2.49. Thus, 184 participants were needed using the formula for a two-sample mean comparison, with a power of 90% and a two-sided *α* of 0.05.

Continuous variables were expressed as mean and standard deviation, while categorical variables were expressed as numbers and percentages. Tests of paired *t*-test, independent *t*-test, ANOVA, *χ^2^* test and Fisher’s exact test were used to compare differences between groups. The Poisson regression model was used to analyze the related factors influencing the workload of health education on admission for nurses, and the results are presented as regression coefficient (*β*) with a 95% confidence interval [*CI*]. The multivariable model evaluated variables for which the *p*-value in the univariate analysis was less than 0.10. All tests were two-sided, and a *p* value of <0.05 was considered statistically significant. SPSS software (SPSS version 22.0) was employed for the analysis.

## Results

**Figure 1:**
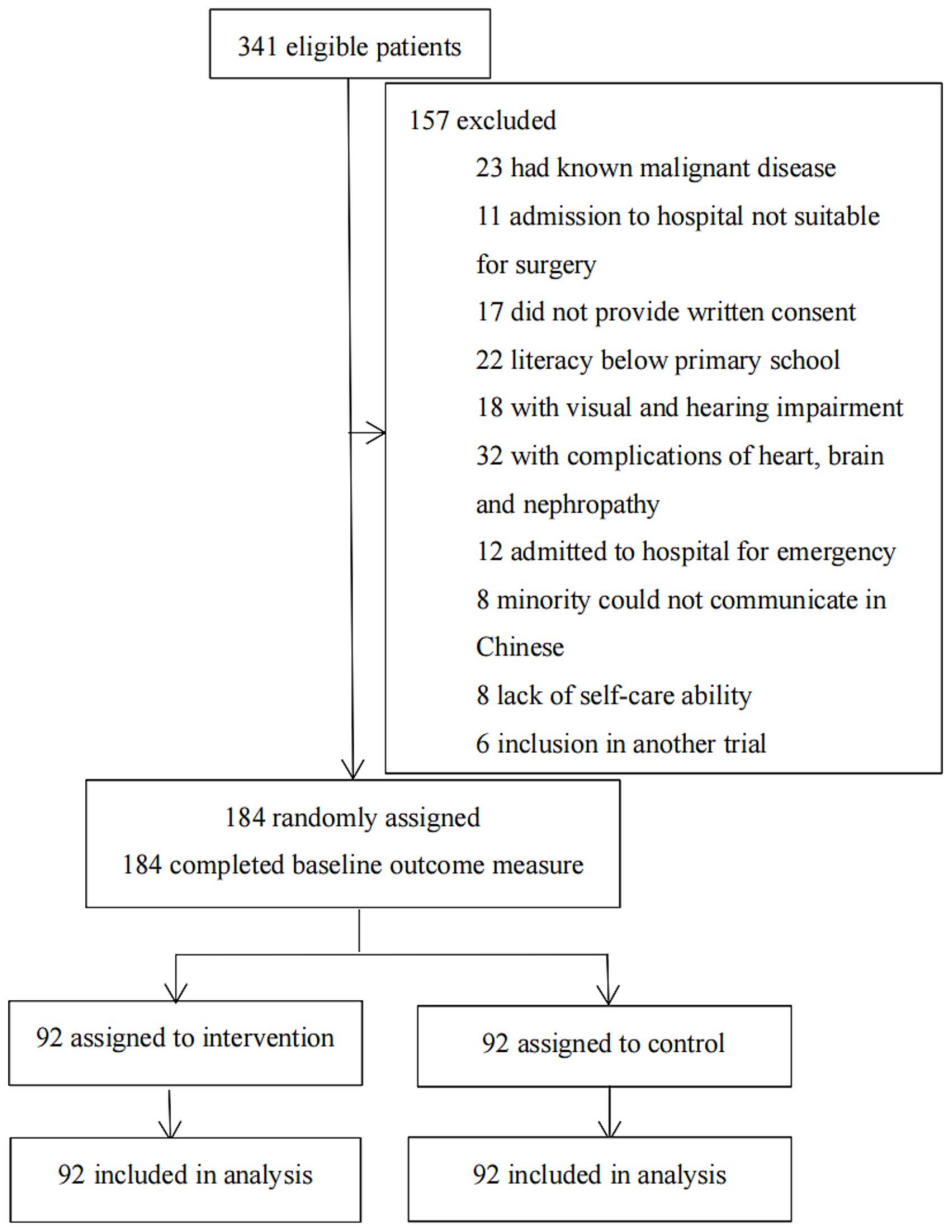
Trial profile.

From July 29, 2019, to June 1, 2022, 341 consecutive patients who were admitted to the Hepatobiliary Surgery Institute at the First Hospital of Lanzhou University were evaluated for eligibility. Following the screening, 157 patients were disqualified (Figure 1). 184 patients who were left were split evenly (1:1) between the multimedia health education group and the standard health education group. The baseline characteristics of the two groups were similar (*p* > 0.05; Table 1). In the intervention group (*n* = 30, 32.6%) more patients underwent open surgery than in the control group (*n* = 25, 27.2%). Additionally, since February 2020, in order to cooperate with the national COVID-19 prevention and control policy, the one-on-one escort system has been strictly implemented in the ward, except in special cases.

**Table 1:** Baseline demographics and clinical characteristics.

Variables	Intervention group (*n* = 92)	Control group (*n* = 92)	*p*
Age (years)	54.39 ± 13.27	53.71 ± 12.37	0.64
Sex, n (%)			0.88
Male	55 (59.8%)	54 (58.7%)	
Female	37 (40.2%)	38 (41.3%)	
Height (cm), mean (SD)	167.85 ± 7.84	170.00 ± 7.74	0.41
Weight (kg), mean (SD)	65.28 ± 10.14	68.20 ± 10.84	0.06
Nation			0.52
Hans	88 (50.6%)	86 (49.4%)	
Minority	4 (49.4%)	6 (50.6%)	
Occupation, n (%)			0.34
Unemployed	5 (5.4%)	4 (4.3%)	
Retiree	23 (25.0%)	17 (18.5%)	
Worker	9 (9.8%)	5 (5.4%)	
Farmer	23 (25.0%)	24 (26.1%)	
Private owner	9 (9.8%)	9 (9.8%)	
Government employee	21 (22.8%)	33 (35.9)	
Student	2 (2.2%)	0 (0.0%)	
Educational Level, n (%)			0.23
Below junior high school	37 (40.2%)	25 (27.2%)	
High school or technical secondary school	11 (12.0%)	20 (21.7%)	
Junior college	28 (30.4%)	27 (29.3%)	
Bachelor’s degree or above	16 (17.4%)	20 (21.75%)	
Diagnosis, n (%)			0.76
Intestinal disease	1 (1.1%)	1 (1.1%)	
Biliary Tract Diseases	44 (44.7%)	50 (54.3%)	
Liver Diseases	8 (8.7%)	4 (4.3%)	
Cancer	29 (31.5%)	28 (30.4)	
Gastric diseases	1 (1.1%)	1 (1.1%)	
Pancreatic Diseases	4 (4.3%)	6 (6.5%)	
Other	5 (5.4%)	2 (2.2%)	
Patients’ anxiety level on admission, mean (SD)	43.47 ± 11.39	43.77 ± 13.09	0.87
Marital status, n (%)			0.16
Unmarried	3 (3.3%)	0 (0.0%)	
Married	87 (94.6%)	88 (95.7%)	
Divorced (Widowed)	2 (2.2%)	4 (4.3%)	
Atopy history, n (%)			1.00
Yes	12 (13.0%)	12 (13.0%)	
No	80 (87.0%)	80 (87.0%)	
Past medical history, n (%)			0.42
Yes	24 (26.1%)	29 (31.5%)	
No	68 (73.9%)	63 (68.5%)	
Operation method, n (%)			0.42
Minimally Invasive Surgery	62 (67.4%)	67 (72.8%)	
Open surgery	30 (32.6%)	25 (27.2%)	
Medical payment method, n (%)			0.66
Urban employees’ basic medical insurance	51 (55.4%)	57 (62.0%)	
Medical insurance for rural residents	31 (33.7%)	27 (31.5%)	
Commercial insurance	10 (10.9%)	8 (8.7%)	
Living environment, n (%)			0.61
City	68 (73.9%)	71 (77.2%)	
Countryside	24 (26.1%)	21 (22.8%)	
Family income (yuan/month), n (%)			0.82
≤3,000	13 (14.1%)	15 (15.8%)	
3,000–5,000	20 (21.7%)	17 (20.1%)	
≥5,000	59 (64.1%)	60 (64.1%)	
Number of caregivers (person), n (%)			0.31
1	74 (80.4%)	79 (85.9%)	
2	18 (19.6%)	12 (13.0%)	
3	0 (0.0%)	1 (1.1%)	
Age of caregivers (years), n (%)			0.40
≤29	4 (4.3%)	9 (9.8%)	
30–39	23 (25.0%)	23 (25.0%)	
40–49	36 (39.1%)	38 (23.9%)	
≥50	29 (31.5%)	22 (23.9%)	

The findings revealed that using multimedia health education can shorten the time nurses spend on health education during patient admissions [15.21 (0.63) vs. 16.94 (3.96), *p* < 0.001] and reduce the number of times patients and their families spend seeking health education information [1.21 (0.62) vs. 2.73 (0.92), *p* < 0.001]. Additionally, the control group’s nurses had to repeatedly explain admission related information to the patients and their families [3.25 (0.89) vs. 1.21 (0.62), *p* < 0.001].

To create satisfaction questionnaires, previous research was consulted (18–20). A team of four experts with backgrounds in medicine, nursing, psychology and outcome measurement evaluated the questionnaires before they were used in the trial. The questionnaire of patients and their families consisted of 11 questions with a total score ranging from 11 (dissatisfaction) to 55 (high satisfaction). The Cronbach’s coefficient (*α*) of this questionnaire was 0.927, expert validity (scale-level content validity index) was 0.93, and Kaiser Meyer Olkin (KMO) was 0.929 (See Supplementary 1). The satisfaction questionnaire for doctors and nurses consisted of 15 questions with a total score ranging from 15 (dissatisfaction) to 75 (high satisfaction). The Cronbach’s coefficient (*α*) of this questionnaire was 0.915, expert validity (scale-level content validity index) was 0.95, and Kaiser Meyer Olkin (KMO) was 0.886 (See Supplementary 2). The results of satisfaction scores showed that use of multimedia health education did not lower the satisfaction levels of patients [53.35 (2.09) vs. 47.86 (5.00), *p* < 0.001], family members [53.35 (2.28) vs. 47.86 (4.53), *p* < 0.001], nurses [73.46 (2.36) vs. 67.16 (5.52), *p* < 0.001] and doctors [73.33 (2.40) vs. 68.07 (4.92), *p* < 0.001] regarding health education (Table 2).

**Table 2:** Main outcomes of health education on admission.

Variables	Intervention group	Control group	*p*
The duration of health education on the day of admission per nurse (min), mean (SD)	15.21 ± 0.63	16.94 ± 3.96	<0.001
Times of health education for patients on the day of admission, mean (SD)	1.21 ± 0.62	3.25 ± 0.89	<0.001
Inquiry times on health education content of patients’ family members, mean (SD)	1.21 ± 0.62	2.73 ± 0.92	<0.001
Satisfaction scores of nurses about health education, mean (SD)	73.46 ± 2.36	67.16 ± 5.52	<0.001
Satisfaction scores of patients about health education, mean (SD)	53.35 ± 2.09	47.86 ± 5.00	<0.001
Satisfaction scores of family members about health education, mean (SD)	53.35 ± 2.28	47.86 ± 4.53	<0.001
Satisfaction scores of doctors about health education, mean (SD)	73.33 ± 2.40	68.07 ± 4.92	<0.001

The Zung Self-Rating Anxiety Scale (SAS) (21) was used to assess patient anxiety levels before and after receiving health education (See Supplementary 3). This scale has high internal consistency in this study sample (*α* = 0.92, 95% CI 0.88–0.95). At admission, there was no difference in anxiety scores between the two groups [43.47 (11.39) vs.43.77 (13.09), *p* = 0.87]. The anxiety scores of the two groups decreased following health education, but there was no statistically significant difference between the two groups [9.20 (6.20) vs. 7.76 (6.50), *p* = 0.13]. According to subgroup analysis, there was a statistically significant difference between anxiety scores of male patients before and after health education [9.40 (6.19) vs. 6.07 (4.31), *p* < 0.001], junior college students [9.89 (6.38) vs. 6.55 (4.46), *p* = 0.03], and patients having open surgery [9.27 (6.97) vs. 5.52 (6.00), *p* = 0.041] (Table 3).

**Table 3:** Anxiety levels before and after health education on admission.

Variables	Intervention group				Control group				*p*
	Before	After	*B-A*	*p*	before	after	*B-A*	*p*	
Total anxiety level	43.47 ± 11.39	34.27 ± 12.46	9.20 ± 6.20	<0.001	43.77 ± 13.09	36.01 ± 13.80	7.76 ± 6.50	<0.001	0.13
Sex				0.70				<0.001	0.06
Male	44.84 ± 11.60	35.44 ± 12.51	9.40 ± 6.19		43.63 ± 13.96	37.56 ± 14.38	6.07 ± 4.31		<0.001
Female	41.43 ± 10.92	32.54 ± 12.36	8.89 ± 6.28		43.97 ± 11.93	33.82 ± 12.81	10.16 ± 8.19		0.45
Educational Level				0.75				0.54	0.43
Below junior high school	45.84 ± 11.65	36.57 ± 13.26	9.27 ± 6.60		43.04 ± 9.83	33.76 ± 11.83	9.28 ± 7.73		0.99
High school or technical secondary school	46.82 ± 12.72	39.36 ± 13.92	7.45 ± 5.30		46.50 ± 14.12	39.75 ± 13.82	6.75 ± 6.34		0.76
Junior college	42.43 ± 10.74	32.54 ± 11.70	9.89 ± 6.38		42.41 ± 13.79	35.85 ± 14.20	6.55 ± 4.46		0.03
Bachelor’s degree or above	37.50 ± 9.14	28.50 ± 8.42	9.00 ± 5.76		438.0 ± 15.11	35.30 ± 16.09	8.50 ± 7.24		0.82
Living environment				0.99				0.24	0.37
City	42.28 ± 10.55	33.09 ± 11.16	9.19 ± 6.15		43.63 ± 13.85	36.31 ± 14.17	7.32 ± 5.97		0.72
Countryside	46.83 ± 13.16	37.63 ± 15.35	9.21 ± 6.46		44.24 ± 10.41	35.00 ± 12.75	9.24 ± 8.02		0.10
Operation method				0.94				0.04	0.20
Minimally Invasive Surgery	39.77 ± 8.94	30.61 ± 9.69	9.16 ± 5.85		42.52 ± 11.88	33.93 ± 12.52	8.59 ± 6.52		0.61
Open surgery	51.10 ± 12.23	41.83 ± 14.22	9.27 ± 6.97		47.12 ± 15.68	41.60 ± 15.71	5.52 ± 6.00		0.041

Before: Patients’ anxiety level before health education; After: Patients’ anxiety level after health education; B-A: Before-After.

Additionally, multimedia health education does not raise the frequency of adverse events in comparison to standard health education (*p* > 0.05; Table 4).

**Table 4:** Adverse events during hospitalization.

Variables	Intervention group	Control group	*p*
Incidence of catheter slippage, n (%)			1.00
Yes	1 (1.1%)	2 (2.2%)	
No	91 (98.9%)	90 (97.8%)	
Incidence pressure ulcer, n (%)			1.00
Yes	0 (0.0%)	1 (1.1%)	
No	92 (100%)	91 (98.9%)	

The results of a univariate analysis indicated that factors influencing nurses’ workload include nation [95% CI: 0.00–0.82, *p* = 0.048], high school or technical secondary school education [95% CI: 0.07–0.64, *p* = 0.014], sequence of patients of the admission day [95% CI: 0.03–0.23, *p* = 0.013] and healthy education methods [95% CI: 1.12–1.68, *p* < 0.0001]. The results of multivariate regression analysis revealed that factors influencing nurses’ workload included healthy education method [95% CI: 1.09–1.67, *p* < 0.0001]. Patients who received oral health education had 1.38 more inquiries than those who received multimedia health education (Table 5).

**Table 5:** Poisson regression analysis of the influence factors of the health education workload on admission.

Variables	Univariable analysis			Multivariable analysis		
	Coefficients	95%CI	*p*	Coefficients	95%CI	*p*
Age	0.00	−0.01, 0.01	0.899			
Sex (male vs. female)	0.09	−0.14, 0.32	0.449			
Nation (Minority vs. Hans)	0.41	0.00, 0.82	0.048	0.42	−0.03, 0.86	0.068
Educational level						
Below junior high school	0			0		
High school or Secondary Special School	0.35	0.07, 0.64	0.014	0.25	−0.06, 0.56	0.109
College degree and above	0.21	−0.06, 0.49	0.128	0.13	−0.16, 0.43	0.382
Past medical history	0.14	−0.09, 0.38	0.232			
Operation method(Open vs. Minimally Invasive)	0.04	−0.20, 0.28	0.763			
Medical payment method(medical insurance vs. Self funded)	−0.05	−0.41, 0.32	0.793			
Living environment(Countryside vs. City)	0.12	−0.13, 0.37	0.328			
Age of caregivers						
≤29	0					
30–39	−0.33	−0.77, 0.10	0.135			
40–49	−0.23	−0.64, 0.18	0.277			
≥50	−0.23	−0.66, 0.19	0.283			
Nurses’ work years	−0.01	−0.03, 0.01	0.212			
Sequence of patients on the admission day	0.13	0.03, 0.23	0.013	−0.03	−0.14, 0.08	0.641
Healthy education methods	1.40	1.12, 1.68	<0.0001	1.38	1.09, 1.67	<0.0001

**Figure 2:**
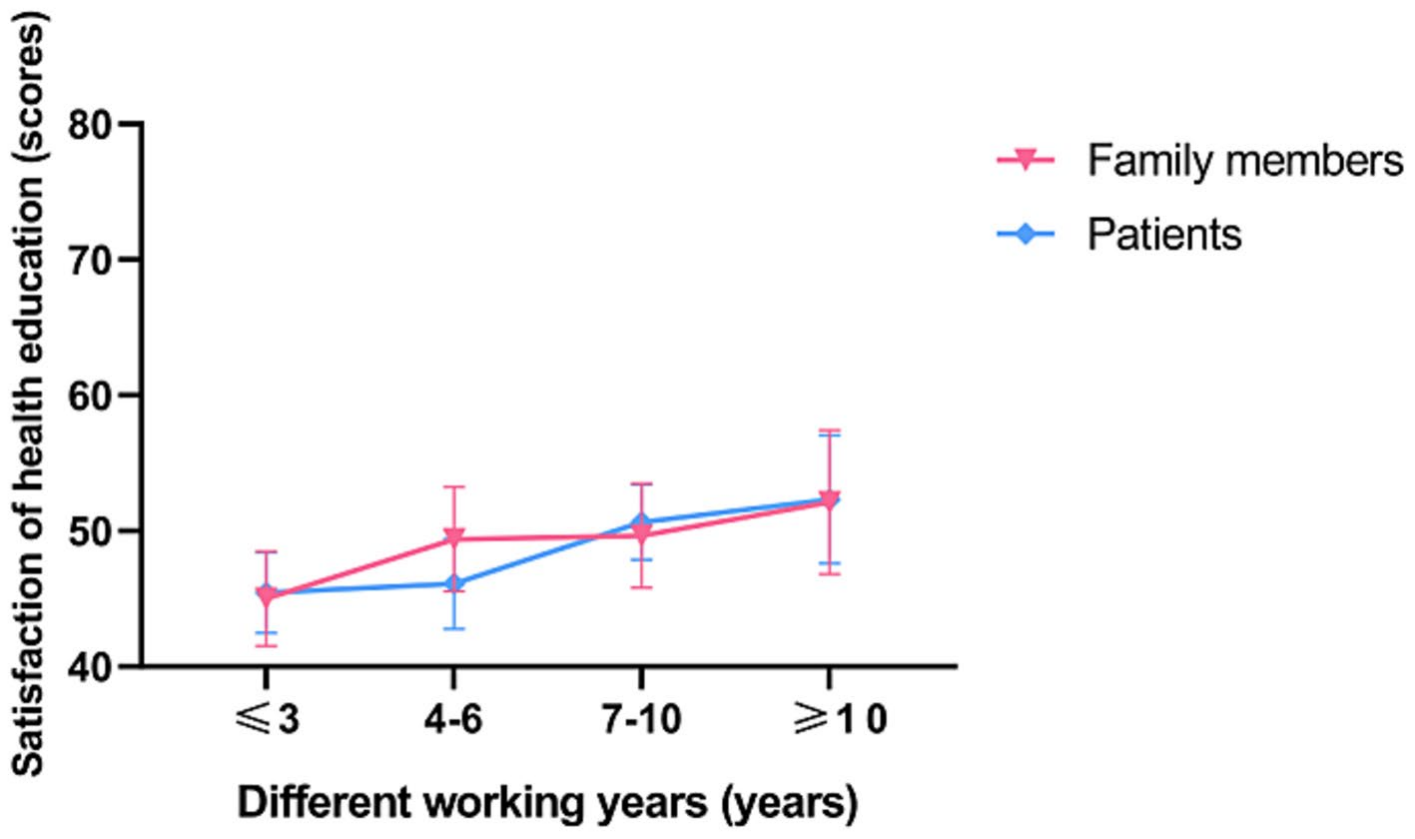
The satisfaction of patients and family members with the health education of nurses with different working years in the control group.

**Figure 3:**
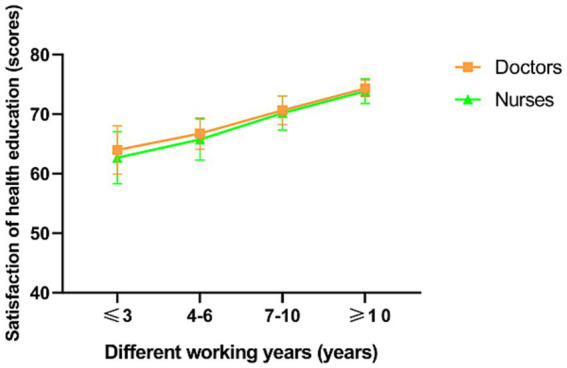
The satisfaction of doctors and nurses with the health education of nurses with different working years in the control group.

**Figure 4:**
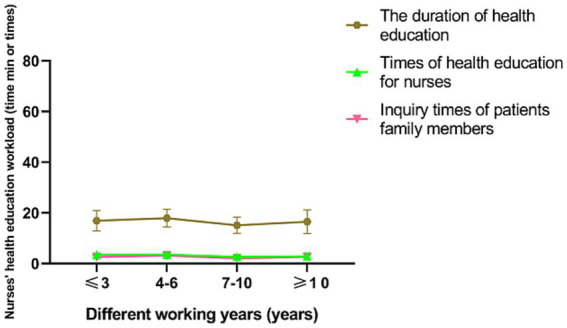
The workload of nurses of health education in the control group with different working year.

**Figure 5:**
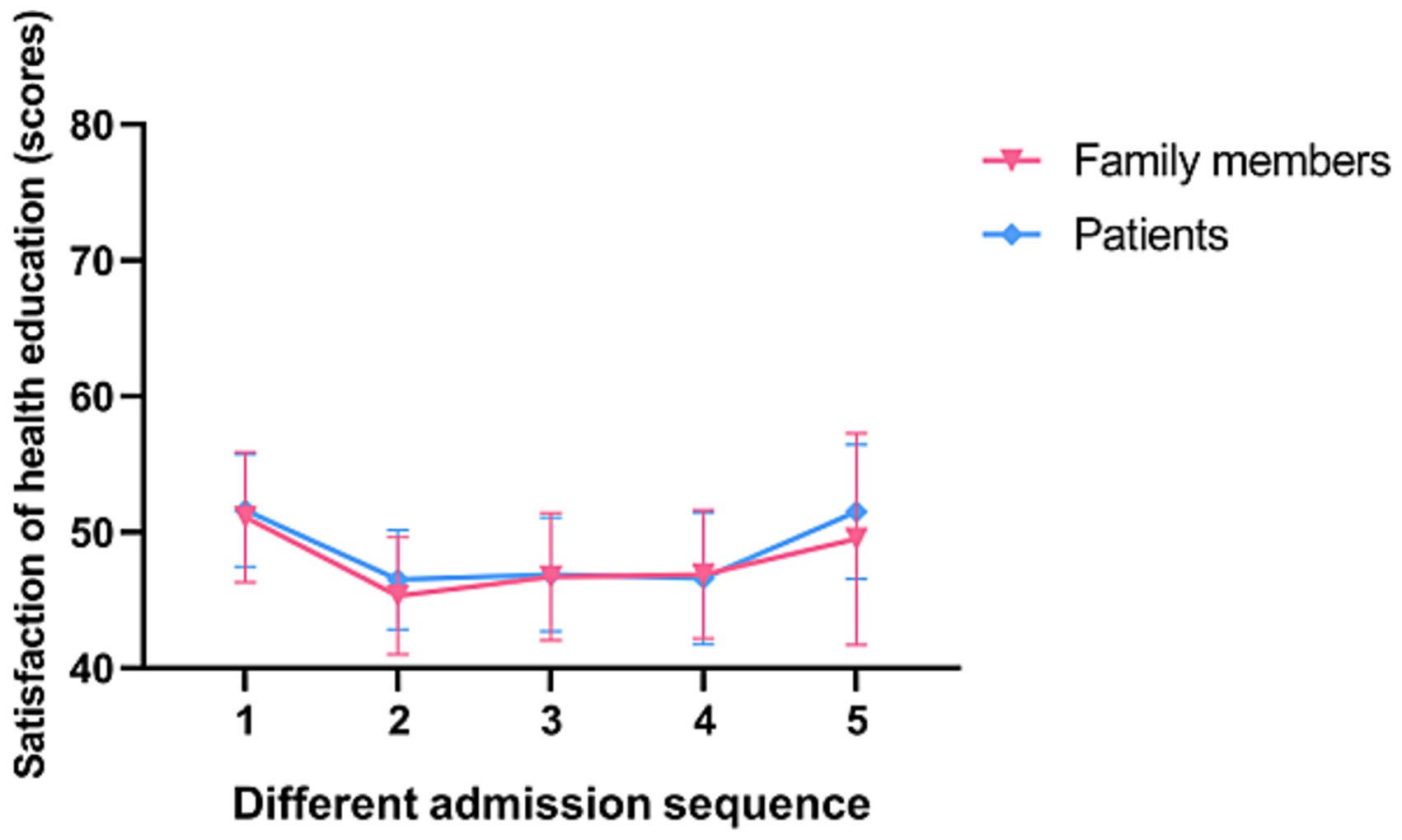
The satisfaction of patients and family members of different admission sequences for health education in the control group.

In a subgroup analysis, doctors, nurses, patients and their families were found to be more satisfied with senior nurse health education (*p* < 0.001; Figures 2, 3). However, there was no significant difference in the duration of health education between nurses with high seniority and those with low seniority (*p* = 0.20). Senior nurses took less time than junior nurses to complete health education for patients and to inquire about the health education of patient family members (*p* < 0.001; Figure 4). The satisfaction with nurse health education was also higher among patients and their families who had been hospitalized earlier in the same shift (Figure 5).

## Discussion

Health information sharing with patients is an important component of treatment plans, which is required and obligated. This information must also be clear, impartial and appropriate. The focus of information must be on the serious risks associated with interventions, any potential alternative strategies and the effects of rejection (22).

Postoperative complications and patient anxiety levels can be decreased with good health education (4, 13, 14, 23, 24). However, whether multimedia health education can significantly reduce the workload of nurses has not been studied yet. In our study, we found that using multimedia health education can shorten the time nurses spend on health education during patient admissions and reduce the number of times patients and their families spend seeking health education information (*p* < 0.001). Our study’s results were consistent with the results of Chang et al.’s study (25) that fewer hours were spent by nurses on health education for the multimedia CD group in comparison with the printed material group. Furthermore, the use of multimedia health education did not lower the satisfaction levels of doctors, nurses, patients, and their families regarding health education; in fact, it increased their satisfaction (*p* < 0.001). Most surgical patients experience varying degrees of psychological stress during the perioperative period (26, 27); good health education can significantly reduce the perioperative patient fear and pain caused by surgical trauma and achieve disease prevention (27–29), as evidenced by high satisfaction scores of patients in the multimedia video health education group. Our study’s findings align with previous research (18, 19), indicating that alternative health education approaches tend to outperform oral health education in terms of patient satisfaction.

In our study, we discovered that the anxiety level of patients included in the study was low and that there was no significant change in the anxiety score following health education (*p* = 0.13). The results of this study were different from the results of Wang Y et al.’s study (14), mainly because patients with serious complications were excluded from this study. However, when compared to female patients, the anxiety score of male patients improved more significantly in the multimedia group (*p* < 0.001). This might be the case because stress may affect male and female brains differently. Furthermore, we discovered that patients with open surgery and a junior college degree experienced a greater reduction in anxiety scores (*p* < 0.05), which was statistically significant as compared to the control group.

Medical management includes health education as a crucial component. The use of patient-needs-oriented health education techniques during the perioperative period can not only increase patient interest in health education but also improve their level of disease management (16, 30–34). Our study demonstrated that the incidence of complications was not increased by multimedia health education. The main reason was that the video presentation was easier to understand than the straightforward verbal presentation. Patients can learn more about the process (31) by participating in multimedia health education and by developing disease self-management abilities (32).

There may be significant differences in the content and outcomes of nurse education at various levels as a result of differences in the professional level, language expressive ability and emotional communication ability of nurses (5, 6). Additionally, because senior nurses have more clinical experience than junior nurses (6), our study found that doctors, nurses, patients and their families were more satisfied with the health education provided by senior nurses in the control group than by junior nurses.

Our study also found that with the increase of patients in the same shift, the satisfaction score of patients and their families gradually decreased, which may be related to nurse compassion fatigue and patience consumption. In addition, our study also found that the health education methods were the influencing factors of nurse health education workload. The effect of health education on other postoperative outcomes and the influencing factors of nurses’ workload are subjects suitable for future research.

The implementation of Enhanced recovery after surgery (ERAS) protocols in surgical wards requires patients and their families to have a profound theoretical understanding of the essence of ERAS. Multimedia video health education can enhance patients’ awareness and comprehension of ERAS, thereby improving their qualification rate of ERAS knowledge. Furthermore, this educational approach can elevate patients’ recovery speed and quality to a higher level. In addition, the scarcity of nursing human resources is a prevalent issue faced by most countries presently. Exploring how to address the shortage of nursing personnel through the utilization of information technology, without compromising patients’ healthcare experiences, constitutes a significant area of research. This study provides a reference basis for the practice of the above clinical measures.

Our study also had limitations. The main limitations of this study were as follows: (1) no multicenter study was conducted, and no information based health education was adopted for medical patients; (2) no specific health education methods were implemented for the different diseases and populations; (3) the research results of this study are not reproducible as they require consideration of specific periods in specific countries, the characteristics of the hospital’s level.

In conclusion, our randomized trial demonstrated that multi-media health education could reduce the workload of nurses and enhance patient satisfaction, but not increase complications. It is worth promoting and applying in clinical nursing work.

## Data Availability

The raw data supporting the conclusions of this article will be made available by the authors, without undue reservation.
