# Supplementary material for: Enhancing patient satisfaction and reducing nurse workload: the impact of multimedia health education in a prospective single-center randomized controlled trial
Source: Front Med (Lausanne). 2025 Feb 19;12:1400061. doi: 10.3389/fmed.2025.1400061 (PMC11880280; doi:10.3389/fmed.2025.1400061)
Supplement: Supplementary file 1 [file Table_1.doc]

**Questionnaire on the satisfaction of patients or family members to the health education on admission**

Dear patients or family members:

In order to provide better nursing service for patients, we designed this questionnaire to investigate the relevant knowledge you acquired through health education on admission. Please fill in it according to your own situation truthfully, and choose only one option for each question. All your information on this questionnaire will be anonymous and will not bring you any inconvenience or trouble. Your work will be conducive to the improvement of nursing quality, thanks for your cooperation.

**Basic information**

1.What is the form of health education the nurse take when you on admission ?

□ Verbal instruction □Video introduction

2.Your age：

3.Your gender

□ Male □ Female

4.What’s your marital status?

□ Unmarried □ Married □ Divorced □ Widowed

5.What’s your occupation?

□ Unemployed □ Civil servant □ Teacher □ Peasant □ Worker

□ Individual operator □ Medical worker □ Other

6.What’s your educational background?

□ Junior high school or below □ High school □ Secondary school

□ Junior college □ Undergraduate □ Master degree or above

7.What’s your medical insurance?

□ Self-funded □ By medical insurance

8.What’s your duration of hospitalization

□ 1 week or less □ 1-2 weeks □ 2-3 weeks □ 3-4 weeks □ 4 weeks or above

9.What’s your family level of income(Per person per month)

□ Less than CNY 10,000 □ CNY 10000-20000

□ CNY 20000-30000 □ More than CNY 30000

10.Who is the caregiver duringyour hospitalization

□ Family members □ Nursing workers □ Friends □ Colleagues □ None

**Patient satisfaction**

1.What do you think of the department environment and notice on admission explained by nurses?

Dissatisfactory Highly satisfactory

2.What do you think of the knowledge about your disease explained by nurse

Dissatisfactory Highly satisfactory

3.What do you think of the dietary precautions during your hospitalization explained by nurse

Dissatisfactory Highly satisfactory

4.What do you think of the precautions of daily life and behavior during hospitalization explained by nurse

Dissatisfactory Highly satisfactory

5.What do you think of that if nurse aked your smoking situation and advised you to give up smoking?

Dissatisfactory Highly satisfactory

6.What do you think of the knowledge about rapid recovery explained by nurse

Dissatisfactory Highly satisfactory

7.What do you think of the notices of health examination explained by nurse

Dissatisfactory Highly satisfactory

8.What do you think of the notices of taking medicine explained by nurse

Dissatisfactory Highly satisfactory

9.What do you think of the safety knowledge explained by nurse

Dissatisfactory Highly satisfactory

10.What do you think of the duration of health education on admission you received from nurse

Dissatisfactory Highly satisfactory

11.What do you think of the way of health education on admission

Dissatisfactory Highly satisfactory

12.What else suggestions about our department?
