# Supplementary material for: Enhancing patient satisfaction and reducing nurse workload: the impact of multimedia health education in a prospective single-center randomized controlled trial
Source: Front Med (Lausanne). 2025 Feb 19;12:1400061. doi: 10.3389/fmed.2025.1400061 (PMC11880280; doi:10.3389/fmed.2025.1400061)
Supplement: Supplementary file 2 [file Table_2.doc]

**Questionnaire on the satisfaction of doctors or nurses to nurses’ admission health education**

Dear doctor or nurse：

Thanks for your support and trust for the work of high quality nursing service sincerely. For better medical cooperation and service for patients, there is a need to research your advice about health education on admission by nurse. Please choose it according to your true feelings, thanks for your cooperation.

1.What do you think of the department environment and notice on admission explained by nurses to patients?

Dissatisfactory Highly satisfactory

2.What do you think of the knowledge about patients disease explained by nurse to patients?

Dissatisfactory Highly satisfactory

3.What do you think of the dietary precautions during your hospitalization explained by nurse to patients?

Dissatisfactory Highly satisfactory

4.What do you think of the precautions of daily life and behavior during hospitalization explained by nurse to patients?

Dissatisfactory Highly satisfactory

5.What do you think of that if nurses aked patients smoking situation and advised you to give up smoking?

Dissatisfactory Highly satisfactory

6.What do you think of the knowledge about rapid recovery explained by nurse to patients?

Dissatisfactory Highly satisfactory

7.What do you think of the notices of health examination explained by nurse to patients?

Dissatisfactory Highly satisfactory

8.What do you think of the notices of taking medicine explained by nurse to patients?

Dissatisfactory Highly satisfactory

9.What do you think of the safety knowledge explained by nurse to patients?

Dissatisfactory Highly satisfactory

10.What do you think of the duration of health education on admission patients received from nurse?

Dissatisfactory Highly satisfactory

11.What do you think of the way of health education on admission?

Dissatisfactory Highly satisfactory

12.What do you think of the service attitude of nurses?

Dissatisfactory Highly satisfactory

13.What do you think of the professional knowledge of nurses?

Dissatisfactory Highly satisfactory

14.What do you think of the nurse's mastery of the patient's condition?

Dissatisfactory Highly satisfactory

15.What do you think of the nurse's professional image.?

Dissatisfactory Highly satisfactory

16.What else suggestions to our department?
