# Supplementary material for: Enhancing patient satisfaction and reducing nurse workload: the impact of multimedia health education in a prospective single-center randomized controlled trial
Source: Front Med (Lausanne). 2025 Feb 19;12:1400061. doi: 10.3389/fmed.2025.1400061 (PMC11880280; doi:10.3389/fmed.2025.1400061)
Supplement: Supplementary file 3 [file Table_3.doc]

**Zung Self-Rating Anxiety Scale (SAS)**

| Items | | | | | Option | Score |
| --- | --- | --- | --- | --- | --- | --- |
| 1. I feel more nervous and anxious than usual. | A | B | C | D |  |  |
| 2. I feel afraid for no reason at all. | A | B | C | D |  |  |
| 3. I get upset easily or feel panicky. | A | B | C | D |  |  |
| 4. I feel like I'm falling apart and going to pieces. | A | B | C | D |  |  |
| 5. I feel that everything is all right and nothing bad will happen. | A | B | C | D |  |  |
| 6. My arms and legs shake and tremble. | A | B | C | D |  |  |
| 7. I am bothered by headaches, neck and back pain | A | B | C | D |  |  |
| 8. I feel weak and get tired easily. | A | B | C | D |  |  |
| 9. I feel calm and can sit still easily. | A | B | C | D |  |  |
| 10. I can feel my heart beating fast. | A | B | C | D |  |  |
| 11. I am bothered by dizzy spells. | A | B | C | D |  |  |
| 1. I have fainting spells or feel like it. | A | B | C | D |  |  |
| 13. I can breathe in and out easily. | A | B | C | D |  |  |
| 14. I get feelings of numbness and tingling in my fingers & toes. | A | B | C | D |  |  |
| 15. I am bothered by stomach aches or indigestion. | A | B | C | D |  |  |
| 16. I have to empty my bladder often. | A | B | C | D |  |  |
| 17. My hands are usually dry and warm. | A | B | C | D |  |  |
| 18. My face gets hot and blushes. | A | B | C | D |  |  |
| 19. I fell asleep easily and get a good night's rest. | A | B | C | D |  |  |
| 20. I have nightmares. | A | B | C | D |  |  |
| Total score |  | | | | | |
| Scoring: positive scoring questions A, B, C and D are scored as 1, 2, 3 and 4 points; The reverse scoring questions are scored by 4, 3, 2 and 1. Reverse scoring question number: 5, 9, 13, 17, 19. The higher the score, the more serious the symptoms. Generally speaking, those whose total anxiety score is lower than 50 are normal; 50-60 is mild, 61-70 is moderate, and more than 70 is severe anxiety. | | | | | | |
